# A Generic Method for Design of Oligomer-Specific Antibodies

**DOI:** 10.1371/journal.pone.0090857

**Published:** 2014-03-11

**Authors:** Kristoffer Brännström, Malin Lindhagen-Persson, Anna L. Gharibyan, Irina Iakovleva, Monika Vestling, Mikael E. Sellin, Thomas Brännström, Ludmilla Morozova-Roche, Lars Forsgren, Anders Olofsson

**Affiliations:** 1 Department of Medical Biochemistry and Biophysics, Umeå University, Umeå, Sweden; 2 Department of Molecular Biology, Umeå University, Umeå, Sweden; 3 Department of Medical Biosciences, Umeå University, Umeå, Sweden; 4 Department of Clinical Pharmacology and Clinical Neuroscience, Umeå University, Umeå, Sweden; Federal University of Rio de Janeiro, Brazil

## Abstract

Antibodies that preferentially and specifically target pathological oligomeric protein and peptide assemblies, as opposed to their monomeric and amyloid counterparts, provide therapeutic and diagnostic opportunities for protein misfolding diseases. Unfortunately, the molecular properties associated with oligomer-specific antibodies are not well understood, and this limits targeted design and development. We present here a generic method that enables the design and optimisation of oligomer-specific antibodies. The method takes a two-step approach where discrimination between oligomers and fibrils is first accomplished through identification of cryptic epitopes exclusively buried within the structure of the fibrillar form. The second step discriminates between monomers and oligomers based on differences in avidity. We show here that a simple divalent mode of interaction, as within e.g. the IgG isotype, can increase the binding strength of the antibody up to 1500 times compared to its monovalent counterpart. We expose how the ability to bind oligomers is affected by the monovalent affinity and the turnover rate of the binding and, importantly, also how oligomer specificity is only valid within a specific concentration range. We provide an example of the method by creating and characterising a spectrum of different monoclonal antibodies against both the Aβ peptide and α-synuclein that are associated with Alzheimer's and Parkinson's diseases, respectively. The approach is however generic, does not require identification of oligomer-specific architectures, and is, in essence, applicable to all polypeptides that form oligomeric and fibrillar assemblies.

## Introduction

The pathological self-assembly of proteins and peptides into amyloid fibrils is the defining characteristic of a group of more than twenty human diseases, including Alzheimer's disease (AD) and Parkinson's disease (PD) [1]. Although amyloid fibrils are invariably present in the affected individuals, many studies have shown that soluble oligomeric assemblies, which can either precede amyloid formation or represent a stand-alone entity formed in parallel with the fibrils, exert the most potent detrimental physiological effects [2]–[11]. However, these oligomers are transient species and frequently only constitute a very minor fraction as compared to the amyloid and the non-aggregated native and precursor forms of the specific protein or peptide. This significantly complicates characterization of oligomers and their selective therapeutic targeting. Intriguingly, antibodies that specifically target oligomeric species have been isolated [11]–[17]. However, the molecular properties of oligomer-specific antibodies are not well understood, which hinders both directed design as well as optimisation of such antibodies. There is, therefore, an urgent need for a method that can be used to consistently and reliably design oligomer-specific antibodies.

Antibodies having the ability to identify structures exclusively present on oligomeric assemblies have previously been demonstrated [11], [13], [14], [18]–[27]. The term “oligomer” can however, be applied to assemblies ranging from a dimer to much larger protofibrillar structures [28]. Due to this inherent heterogeneity, and the lack of *a priori* structural information, directed design of oligomer-specific antibodies is not straightforward and is frequently dependent on stochastic events. These limitations hamper development in the field. We have previously shown that the multivalent architecture of IgM antibodies, having 10 independent binding sites, can be used as a selective binder for oligomers due to the exposure of multiple epitopes on the oligomeric assemblies [29]. However, the IgM isotype cannot be recombinantly expressed or genetically modified, and this hampers both its characterisation and its potential therapeutic use.

In the present work, we show how a simple divalent binder such as antibodies of the IgG isotype is an interesting alternative. In contrast to the multivalent IgM a divalent interaction importantly facilitate the factors required for oligomer-specificity to be determined in a quantitative manner. Through characterising of a spectrum of monoclonal antibodies, having significantly different properties, the concept of oligomer-specificity is outlined and we demonstrate how different parameters affect the efficiency of selectively binding to oligomers. We expose how the ability to bind oligomers is affected by the monovalent affinity and the turnover rate of the binding and, importantly, also how oligomer specificity is only valid within a specific concentration range. We have specifically applied the method to identify oligomer-specific monoclonal antibodies targeting the amyloid β peptide (Aβ) and α-synuclein that are associated with AD and PD, respectively. The approach is however generic and applicable to all polypeptides that form oligomeric and fibrillar assemblies.

## Results

### Step 1: Discriminating between oligomers and amyloid fibrils

The definition of an oligomer-specific antibody implies that it does not react with the fibrillar or monomeric counterparts of the same protein or peptide. The first step in the present method is to find an epitope which exclude binding of the antibody to the fibrillar form of the polypeptide. To accomplish this, we use the structural differences between the fibrillar and oligomeric structures and through identification of a cryptic epitope that is exclusively buried within the fibrillar architecture, discrimination can be acquired.

### Identifying epitopes having different exposure on Aβ oligomers and Aβ fibrils

For Aβ, the N-terminal residues have previously been shown to be differently exposed between the fibrillar and oligomeric forms [30], [31]. Mice were immunised with the Aβ_1–42_ and hybridomas were generated according to standard procedures. The antibody response in mice upon immunisation with Aβ frequently results in antibodies that target the Aβ_(3–10)_ epitope, and a number of these have also been reported in the literature [32]. Using a dot blot technique – in which an equal amount of fibrils and monomers from the Aβ_1–42_ are applied to the membrane – we concluded that the region spanning residue 3–10 in a nice manner can be used to differentiate between the fibrillar and oligomeric forms. From the initial pool of clones targeting Aβ, two monoclonal IgG antibodies denoted mAB-O and mAB-M, that both bind within the Aβ_(3–10)_ region, but also in a good way illustrate the subsequent requirements for oligomer-specificity further presented below, were selected.

**Figure 1:**
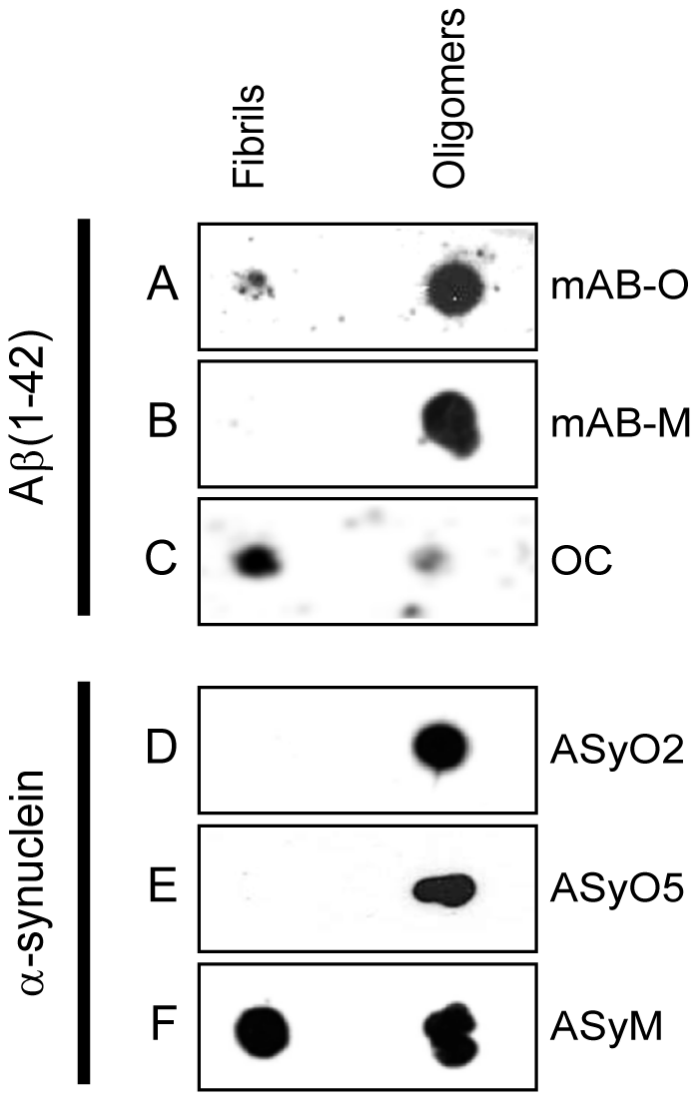
Dot blot enables probing for structural differences between fibrils and oligomers. An equal amount of fibrils and oligomers from either Aβ_(1–42)_ or α-synuclein, as indicated in the figure, were applied to a nitrocellulose membrane. Binding specificity of (A) mAB-O, (B) mAB-M, (C) Aβ fibril-specific OC antibody,, (D) ASyO2, (E) ASyO5, and (F) ASyM.

A dot blot analysis of mAB-O and mAB-M is shown in Figure 1 A and B and illustrates the preferential binding to oligomers and how both antibodies are impaired in binding to the fibrillar fold. The conformational dependence and fibril specificity of the monoclonal antibody OC [33] was used to verify the presence of Aβ fibrils (Fig. 1C). The different morphologies of oligomeric and fibrillar Aβ_(1–42)_ were also confirmed by atomic force microscopy (AFM) (Figure S1).

### Identifying epitopes having different exposure on α-synuclein oligomers and α-synuclein fibrils

There is only limited knowledge of the structural properties of α-synuclein's oligomeric form, so multiple antibodies where generated using the complete 140-residue sequence of α-synuclein as the antigen. Using the dot blot technique - where equal amounts of α-synuclein fibrils and monomers were applied to a membrane followed by screening for selective binding to oligomers - we identified a comparatively broad region in the C-terminal part of the protein (from Gly111 to Ala140) that is exposed in the oligomeric form but buried within the fibrillar form.

Two different monoclonal antibodies having the IgG isotype, that both bind within the Gly111–Ala140 region of α-synuclein, but also in a nice manner illustrate parameters important for oligomer-specificity further presented below were selected. These antibodies, denoted ASyO2 and ASyO5, bind to the Glu131–Ala140 and Gly111–Tyr125 regions, respectively, and both readily bind to α-synuclein oligomers but not to α-synuclein fibrils (Fig. 1 D and E).

A third α-synuclein–specific antibody, denoted ASyM, binds to the N-terminal stretch spanning Met1–Val15, but this is a sequence that does not effectively discriminate between oligomers and fibrils. This antibody was used as a control to expose the equal amounts of fibrils and oligomers on the membrane (Fig. 1F) as well as to further expose how differences in binding kinetics in a dramatic manner may affect oligomer specificity, described below.

The structures of the fibrillar and oligomeric samples of α-synuclein were also verified using AFM (Figure S1).

### Step 2: Discriminating between monomers and oligomers

In the second step of the method, the ability to discriminate between oligomers and monomers is evaluated. This step harnesses the effect of avidity, which is defined as a synergistic increase in binding strength when a multivalent receptor binds to a multivalent antigen. The impact of avidity and its consequences for oligomer-specificity is exposed through a comparative analysis of the above selected spectrum of antibodies, which are all divalent and covers a broad range of monovalent affinities. Through this comparison the potential use of a simple divalent interaction to discriminate between monomers and oligomers is revealed and a method which enables design of oligomer-specific antibodies is uncovered.

The concept of avidity applies to all antibodies having a valency higher than one. According to the fundamental properties of molecular binding between a receptor and its ligand, the level of saturation is dependent on the highest concentration of either the ligand or the receptor. At concentrations significantly above K_D_, an interaction will approach full saturation according to Equation 1 (defined in Methods). Through the effect of avidity, the interaction with a multivalent target will result in a higher binding strength. For a divalent antibody, this will result in two different K_D_ values. At concentrations between these two K_D_ values (K_Ddiv_ and K_Dmono_), binding to the oligomers will be favoured. Consequently, a difference in saturation level between a monovalent interaction and its divalent counterpart is only valid within a specific concentration range. The size of this range is dependent on the monovalent affinity and the increase in binding strength due to avidity.

The present spectrum of antibodies, including both the anti-Aβ and the anti-α-synuclein antibodies, illustrates well how different monovalent K_D_ values are reflected within their increased ability to bind oligomeric assemblies compared to monovalent antigens at different antibody concentrations. The monovalent binding affinities of all antibodies were acquired using standard surface plasmon resonance (SPR) techniques where immobilized antibodies are probed against soluble monomers (all SPR sensograms are shown in Figure S2 of the Supporting Information). The divalent binding strengths were determined using a solution-based competition assay that is described in detail in the Materials and Methods section. The result of the SPR assay is summarized in Table 1 and shows the monovalent and divalent interaction strength of each antibody as well as the corresponding increase in binding strength given by the K_Dmono_/K_Ddiv_ ratio.

**Table 1:** A summarized table displaying the monovalent and divalent affinities of the evaluated antibodies.

	K_Dmono_	K_off (mono)_ t_(1/2)_ (s)	K_Ddiv_	K_Dmono_/K_Ddiv_
mAB-O	36 µM	0.5 s	23 nM	1500
mAB-M	32 nM	495 s	50 pM	640
ASyO2	2000 nM	20 s	3 nM	690
ASyO5	500 nM	20 s	0.79 nM	625
ASyM	1.7 nM	6300 s	n.d	n.d

The ratio between K_Dmono_/K_Ddiv_ illustrates the potentiating increase in binding strength as a result of avidity and the monovalent dissociation rates, K_off (mono)_ t_(1/2)_, highlights the significant differences in binding kinetics between the different antibodies.

**Figure 2:**
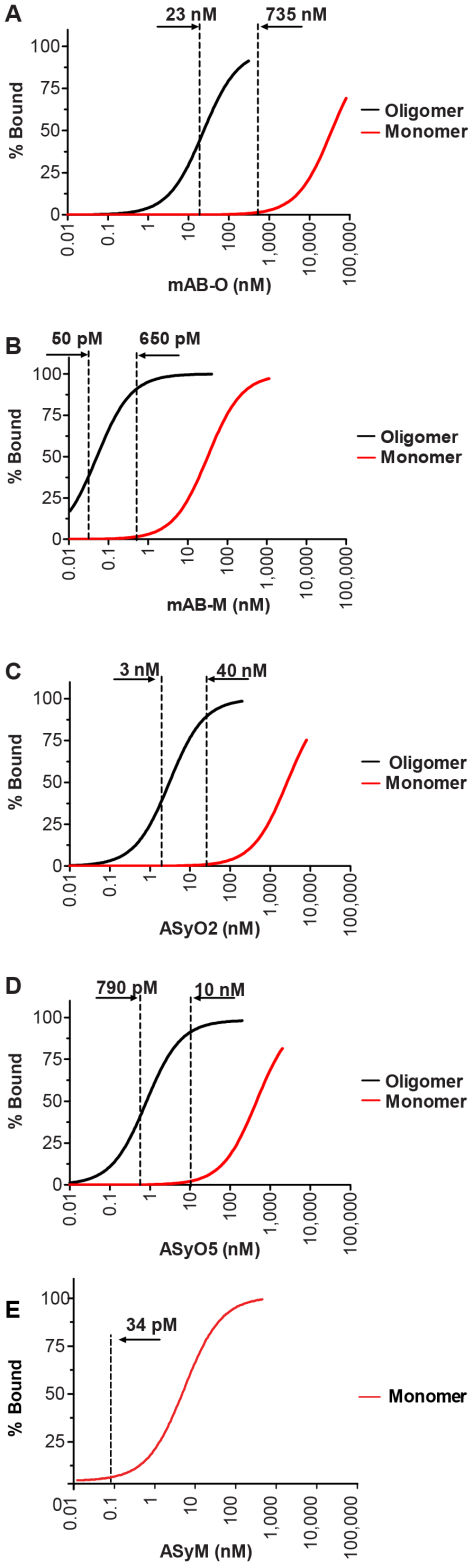
Monovalent and divalent saturation level as a function of concentration. The level of antibody saturation upon binding to oligomers is shown as a black line and binding to monomers is shown as a red line. The vertical dotted lines indicate the concentrations where the saturation level of binding to the oligomer is above 50% while the monovalent saturation level remains below 2%. (A) mAB-O, (B) mAB-M, (C) ASyO2, (D) ASyO5, (E) ASyM.

The difference in saturation level upon binding to oligomers versus monomers as a function of concentration is illustrated in Figure 2A–E. For reasons of comparison, we have illustrated the concentration range for all antibodies where the saturation level for binding to the oligomer is above 50% and less than 2% of the antibodies are bound to the monomer.

mAB-O displays an extremely weak monovalent binding and has a K_Dmono_ corresponding to 36 µM. Upon binding to oligomers, its K_Ddiv_ corresponds to 23 nM and thus displays a more than 1500-fold increase in the binding strength (Fig. 2A). The span between >50% bound oligomer and <2% bound monomer is between 23 nM and 735 nM.

The binding strength of mAB-M is increased 640-fold as a result of avidity. Due to the higher monovalent affinity (32 nM), however, the corresponding concentration range where a saturation level for the oligomer is above 50% while less than 2% of the antibodies are bound to the monomer becomes very narrow and only spans between 50 pM and 650 pM (Fig. 2B). The useful concentration range for mAB-M is, therefore, approximately 1000-fold smaller than for mAB-O.

The potentiating effects of ASyO2 and ASyO5 correspond to 690-fold and 625-fold, respectively, and these antibodies display monovalent affinities corresponding to 2000 nM and 500 nM, respectively. The concentration where 50% binding occurs while the saturation level of binding to the monomer is less than 2% corresponds to 3–40 nM for ASyO2 and 0.79–10 nM for ASyO5 (Fig. 2C and D).

The divalent binding strength of ASyM, which has a K_Dmono_ of 1.7 nM, was too strong to be experimentally determined. A monovalent level of saturation corresponding to 2% for ASyM is, however, already reached at 34 pM (Fig. 2E). Below these results are also presented in a non-logarithmic manner in further described below, that better illustrates how the width of the useful “concentration window” is dramatically increased simply by lowering the monovalent affinity.

### A high turnover rate facilitates rapid identification of the oligomeric species

Antibody efficacy can also be described in terms of turnover rate. This parameter relates to the kinetic properties of the antibody and its ability to screen through a solution and selectively identify oligomers out of a stoichiometric excess of the monomeric counterpart. The turnover rate of binding between, for example, a receptor and a ligand is intimately connected to K_D_, which is defined by the ratio between the association rate constant (k_on_) and the dissociation rate constant (k_off_) (Eq. 2). Consequently, a high k_on_ and k_off_ will generate a rapid rate of turnover and hence the binding will reach equilibrium fast. A slow rate of turnover, on the contrary, will lead to a kinetically impaired antibody and limit the screening capability. Present selection of antibodies will illustrates this effect.

**Figure 3:**
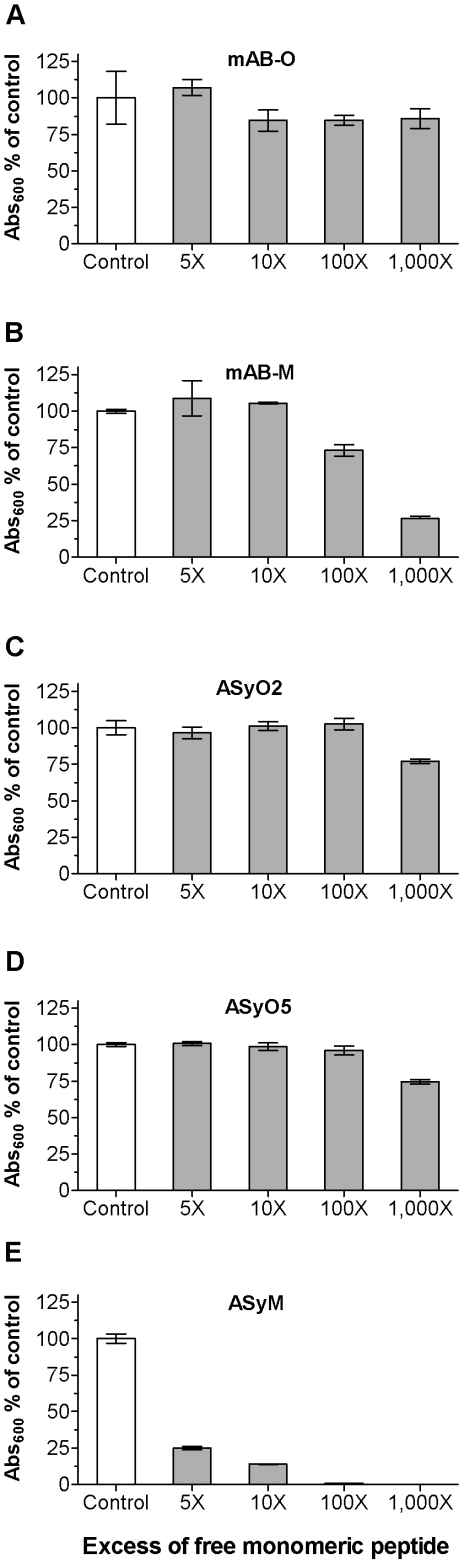
The rate of turnover controls antibody efficacy. Competition ELISA setup showing the efficacy of the antibodies to detect oligomers in the presence of increasing concentrations of monomers. (A) mAB-O. (B) mAB-M. (C) ASyO2. (D) ASyO5. (E) ASyM. The kinetics of binding for each antibody are given in Table 1.

Low-affinity antibodies often have a fast rate of turnover, and the k_off_ is often the parameter that shows the most variation. Therefore, the t_(1/2)_ k_off_ for all investigated antibodies is given in Table 1. To illustrate the importance of turnover rate, we developed an ELISA-based competition assay where oligomers were immobilised on the plate and the corresponding antibodies were added in combination with an increasing amount of free monomeric peptide. mAB-O, which has a very low monovalent affinity and a rapid turnover rate, efficiently detected oligomers even in a 1000-fold excess of free monomers (Fig. 3A). mAB-M, which has a higher monovalent affinity, was partly inhibited above a 100-fold excess of the monomer (Fig. 3B). Both ASyO2 and ASyO5 efficiently identified oligomers in the presence of a 1000-fold excess of monomeric peptides (Fig. 3C and D) while ASyM, having a significantly slower turnover rate, in contrast was effectively inhibited from binding and displayed an essentially stoichiometric level of inhibition as a result of competition with the monomer (Fig. 3E).

### Dot blots for discriminating between monomers and oligomers can be deceptive

**Figure 4:**
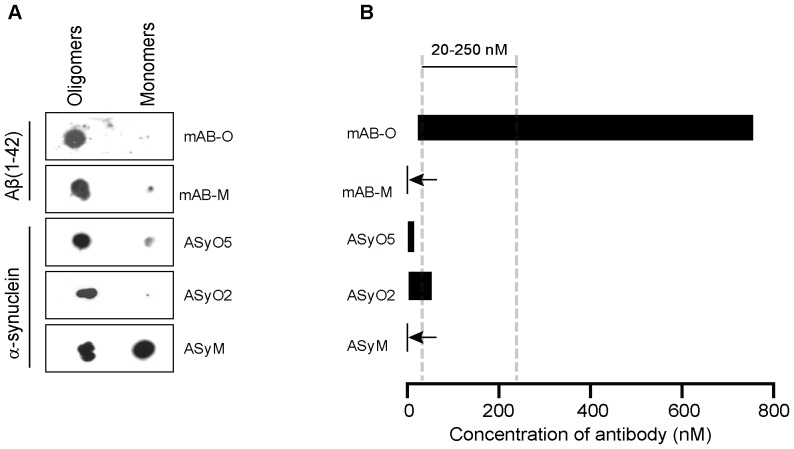
Therapeutic antibody concentrations require very low monovalent affinities to avoid reactivity to monomers. (A)Non-logarithmic illustration of the antibody concentration range where more than 50% of the oligomers are bound while the saturation level to the monomer is below 2%. Note in particular the dramatic difference between mAB-O and mAB-M that both qualify as being oligomer specific according to the widely accepted dot blot technique. The bars corresponding to mAB-M as well as ASyM are indicated with arrows to highlight their appearance. For comparison, the most commonly used range of therapeutic antibody concentrations for AD (20 µM to 250 µM) is indicated in the figure (grey striped vertical bars). (B) Dot blot analysis where equal amounts of Aβ_1–42_ and α-synuclein oligomers and monomers has been applied on a membrane and probed with mAB-O, mAB-M, ASyO5, ASyO2, and ASyM as indicated in the figure.

The dot blot technique – which is frequently used for discriminating between oligomers and monomers – should be highlighted because it might yield deceptive results if not performed properly. The effect of avidity, which applies to all antibodies having a valency higher than one, results in a potentiation in binding strength as a result of two or more simultaneous interactions. This also implies that potentiation in the binding strength will occur if antigens that are immobilized on a surface are separated by an average distance less than the distance between the two antigen-binding sites on the antibody. When using techniques such as dot blot, western blot, or ELISA for the purpose of discriminating between oligomers and monomers, this effect must be considered and the antigens must consequently be diluted accordingly to be separated by an average distance on the membrane higher than the distance between the binding sites on the antibody. The multiple epitopes of an oligomer are close to each other due to the structure of the oligomer but the average distance between immobilized monomers becomes too far to mediate the effect of avidity and the potentiated binding strength. The effect is interpreted as an oligomer-specific interaction. A dot blot of the oligomeric versus monomeric species is illustrated in Fig. 4A. In this context it should however be emphasized that dot blot techniques do not reflect the level of saturation of binding. The ability to remain bound throughout the assay, including the subsequent washing steps, is therefore controlled by the dissociation rate of the antibody. This implies that antibodies having a high affinity, but also a high turnover rate, i.e. a fast k_on_ and a fast k_off_, will not necessarily remain bound throughout the assay. Fig. 4B illustrates the window of concentration where binding saturation to the oligomer is above 50% while binding to the monomer is below 2% for all evaluated antibodies (adapted from the results shown in Fig. 2 but now presented in a non-logarithmic manner). In particular mAB-M, which has a K_D_ of 32 nM, exemplifies the phenomenon where a high level of saturation during the initial incubation passes undetected. During the dot-blot procedure all antibodies are incubated at 25 nM which regarding mAB-M results in a 44% level of saturation. However, due to its comparatively high dissociation rate mAB-M is unable to remain bound throughout the assay and the result is interpreted as oligomer-specific. Within the present experiment only the ASyM antibody, as a result of its slow turnover rate, remains bound throughout the dot blot assay.

### Imunohistochemistry on AD brain tissue

**Figure 5:**
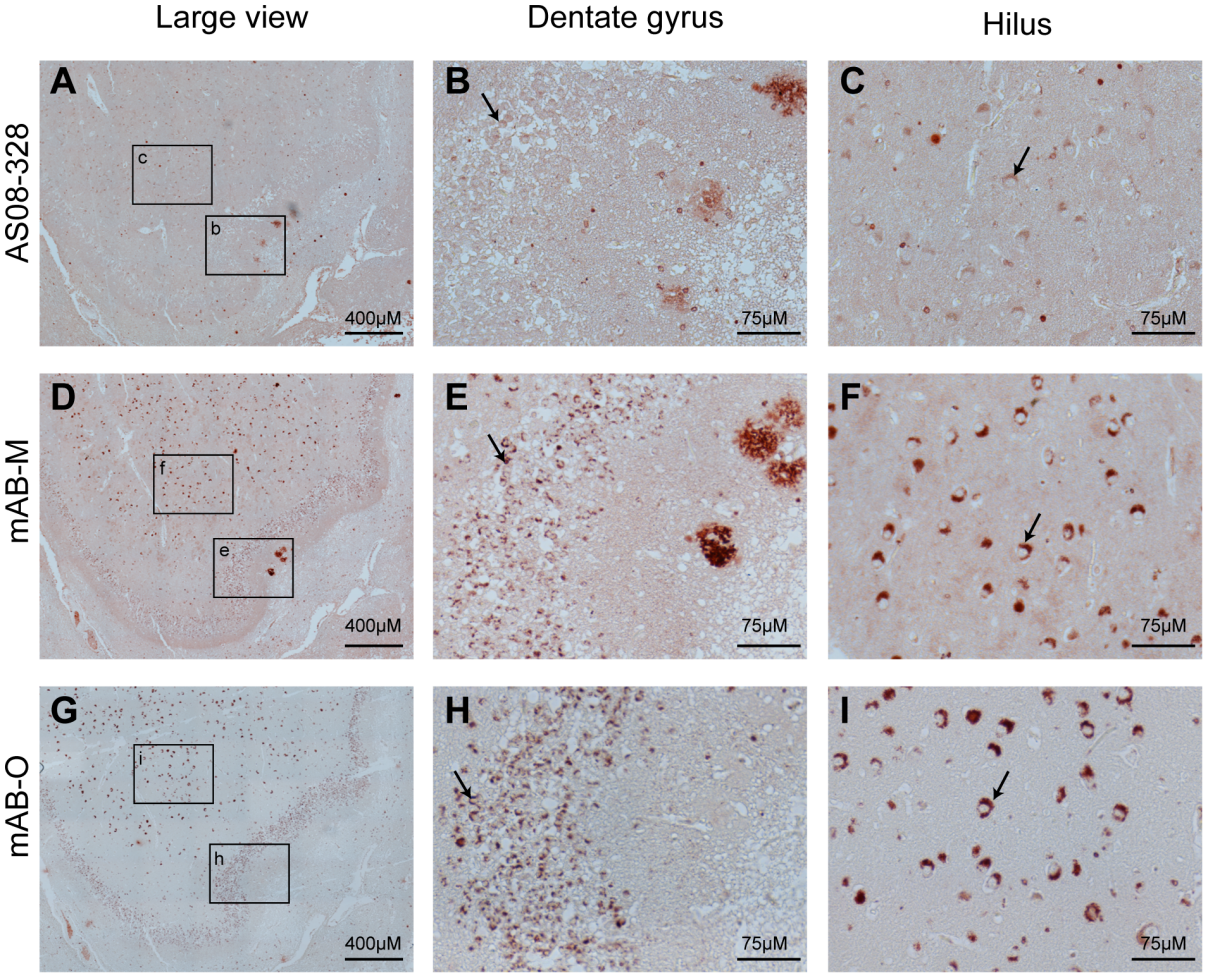
IHC of human hippocampus from an AD-affected individual. IHC was used to illustrate binding specificity and structural preferences of mAB-O and mAB-M upon binding to *ex vivo* material. Hippocampus sections were from a post-mortem AD-affected human brain (A–C) The binding pattern from a polyclonal rabbit anti-Aβ antibody illustrating both plaques as well as an intracellular form of Aβ that is abundant in the hilar neurons. (D–F) The staining pattern of mAB-M in which Aβ plaques near the dentate gyrus (E) are readily stained. (G–I). The binding pattern of mAB-O illustrates an inability to stain Aβ plaques and indicates an alternative structural preference compared to mAB-M. A strong binding to the intracellular form of Aβ is observed in the hilus area. The granular cells of the dentate gyrus (B, E, and H) and the neurons of hilus (C, F, and I) are indicated with arrows and represent the highe magnification images of respective selected areas in A, D and G.

**Figure 6:**
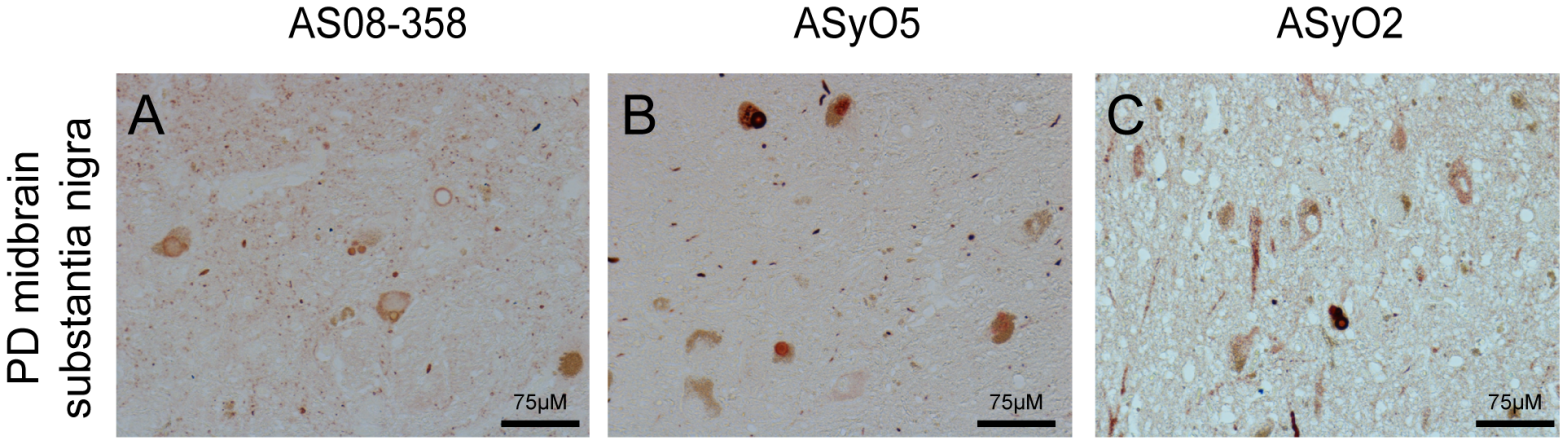
Immunohistochemistry of human midbrain from a PD-affected individual. To probe the specificity and structural preference of ASyO2 towards *ex vivo* α-synuclein assemblies, the mesencephalon (midbrain) of the brain of a human PD patient was analysed. The images represent the substantia nigra area of the midbrain. (A) Polyclonal anti α-synuclein. (B) ASyO5. (C) ASyO2.

For most antibodies, a high binding specificity over non-specific background binding is often essential, it is therefore important to emphazise that a low affinity is not necessarily associated with broad specificity. To illustrate that a high specificity is maintained, as well as to evaluate their binding pattern to *ex vivo* material, the ability of the antibodies created in this study to bind to human brain-derived Aβ and α-synuclein deposits was evaluated using immunohistochemistry (IHC) Figures 5 and 6.

The hippocampus area from a human AD patient was evaluated using polyclonal rabbit anti Aβ antibody (Fig. 5A–C), mAB-M (Fig. 5D–F) and mAB-O (Fig. 5G–I). The results show that the overall background staining of all these antibodies was low (Fig. 5A,D,G). The results also revealed an intriguing discrepancy between mAB-M and mAB-O where mAB-M efficiently bound Aβ plaques but no reactivity could be seen with mAB-O (Fig. 5E and 5H respectively). The reactivity of mAB-M to amyloid plaques consequently pinpoints a discrepancy when compared to the dot blot analysis, shown in Figure 1B. The presence of less condensed protofibrils or diffuse non-fibrillar plaques, having a more open architecture, should be considered as possible explanations. Another reason for this difference might be the fact that the conditions used to prepare the IHC tissue samples are often harsh and could possibly induce exposure of previously buried epitopes on the fibrils.

Nevertheless, the results illustrate how subtle changes in epitope exposure can have profound impacts on the reactivity's of the antibodies. Interestingly all Aβ antibodies, including the polyclonal anti-Aβ antibody (Fig. 5A–C), bound to an intracellular form of Aβ that has been previously described but is not currently associated with disease [34]. The binding to intracellular Aβ was notably stronger for mAB-M (Fig. 5E,F) and mAB-O (Fig. 5H,I) both in granular neurons of dentate gyrus and in hilar neurons compared to the polyclonal antibody (Fig. 5B,C).

### Immunohistochemistry in PD brain tissues

To explore the specificity and binding patterns of ASyO2 and ASyO5, these antibodies were used to probe a tissue sample isolated from the substantia nigra of the brain from a PD patient (Fig. 6). The results show that both ASyO5 and ASyO2 specifically bind to α-synuclein in Lewy bodies and Lewy neurites (Fig. 6B and C) with a staining pattern similar to that of the polyclonal anti-α-synuclein antibody AS08-359 (Fig. 6A). Reactivity to deposited α-synuclein material could indicate the presence of pre-fibrillar structures *in vivo*, but we cannot explicitly rule out the possibility that *in vivo* and *in vitro* fibrils might differ in structure or that the exposure of these epitopes might be caused by the sample preparation and might, therefore, not be fully representative of the situation *in vivo*.

### mAB-O efficiently prevents the cytotoxic effect of Aβ-oligomers

**Figure 7:**
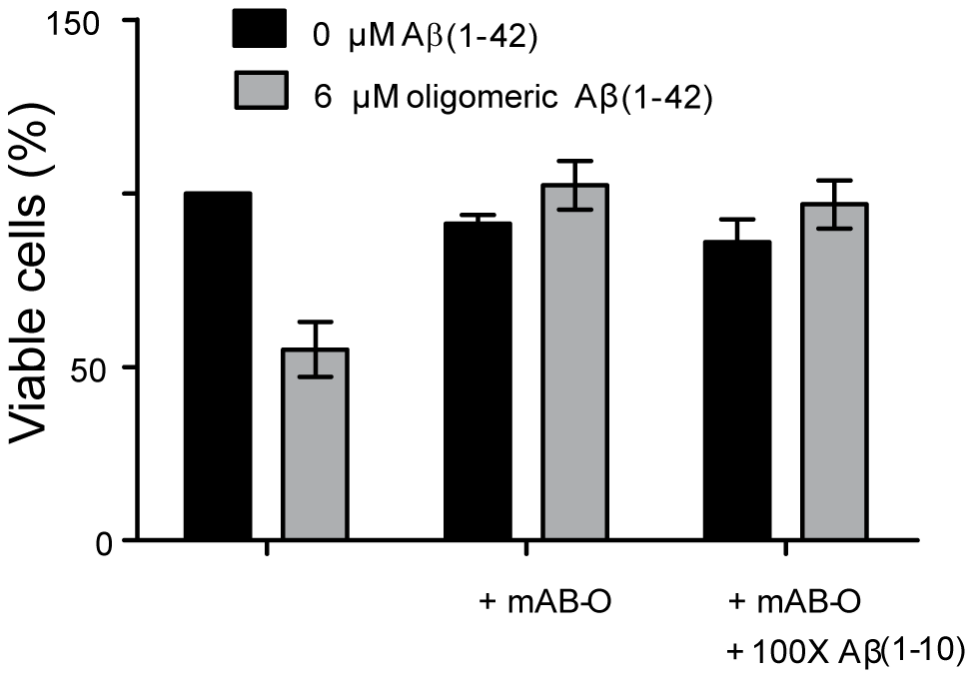
Binding to biologically active oligomers of Aβ_1–42_. Isolated Aβ_1–42_ oligomers exert a cytotoxic effect on SH-SY5Y cells, and the attenuating effect of the oligomer-specific mAB-O antibody was evaluated. Cells exposed to Aβ_1–42_ oligomers are shown with grey bars while controls are depicted in black. Addition of mAB-O in a 1∶5 molar ratio to Aβ_1–42_ fully attenuates the effect. Addition of Aβ_1–10_ monomer at a 100-fold molar excess does not interfere with the attenuating effect.

Oligomer-specific antibodies have therapeutic potential. It is, therefore, important to verify that antibodies having very low monovalent affinities are still able to prevent a cytotoxic effect. Addition of pre-formed Aβ oligomers to the human neuroblastoma cell-line SH-SY5Y exerted a cytotoxic effect, and within 48 hours of incubation cell viability was reduced to 60% (Fig. 7). Addition of mAB-O to the system resulted in an efficient attenuation of the cytotoxic effect (Fig. 7). Notably, the same inhibiting effect could also be obtained in the presence of a 100-fold molar excess of monomeric Aβ_1–10_ peptide. This suggests that the monomeric form of the antigen did not affect antibody binding to the oligomeric form. We were unable to perform the same assay with α-synuclein because the cytotoxic effect from these oligomers is significantly lower and requires significantly higher protein concentrations. The required concentration of antibodies could not be added without unwanted side effects in the cell assay.

### Detection of *ex vivo*-isolated oligomers

**Figure 8:**
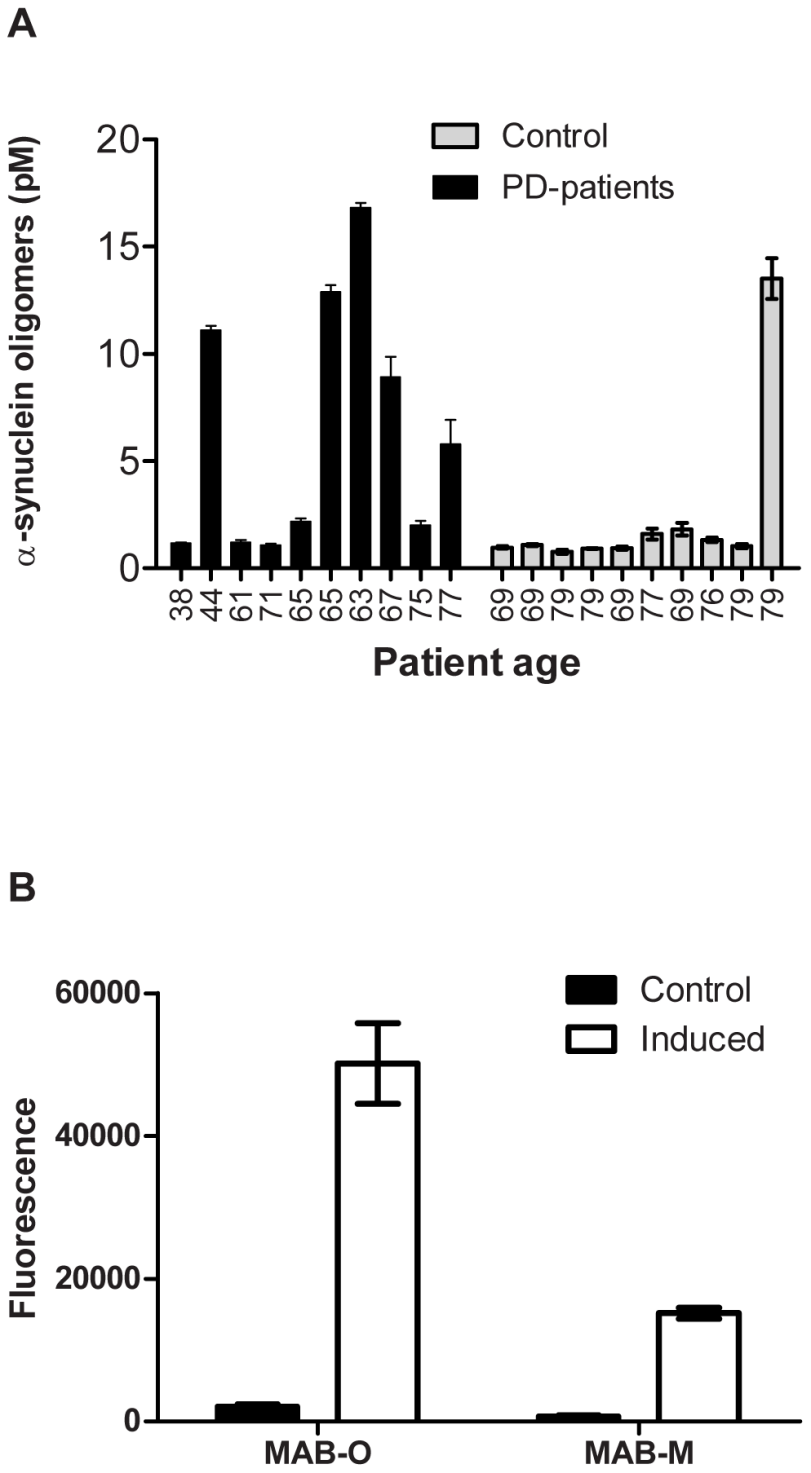
Detection of *ex vivo*-derived oligomeric assemblies. (A) CSF samples from 10 PD patients having a clinical stage of disease around 2 according to the Hoehn and Yahr scale [35] and 10 age-matched controls were probed for the presence of α-synuclein oligomers using a homopaired sandwich ELISA based on the ASyO2 antibody. The age of each individual is given below the corresponding bar. The concentration of α-synuclein oligomers was determined using a standard curve of *in vitro* α-synuclein oligomers based on their monomeric protein concentration. (B) A sandwich ELISA setup using mAB-O and mAB-M to evaluate binding to Aβ oligomers derived from the well-established and previously described 7PA2 cells [38].

Oligomeric assemblies can be found in the cerebrospinal fluid (CSF) of both AD and PD patients at concentrations within the lower picomolar (pM) range. In the present investigation, CSF samples from PD patients were analysed, and all patients were classified as being around stage 2 of the disease according to the Hoehn and Yahr scale [35]. In total, CSF samples from 10 PD patients and 10 age-matched controls were evaluated. Using a homopaired sandwich ELISA based on the ASyO2 antibody, a significant increase in the presence of oligomers was seen in the group of PD patients (Fig. 8A). In accordance with similar investigations, oligomeric α-synuclein could be seen in some controls and there were some individuals with PD who did not have α-synuclein oligomers in their CSF [36], [37].

A similar set of CSF samples was not available for AD during the time of this investigation. Therefore, to evaluate the ability of the anti-Aβ antibodies to capture oligomers produced by a eukaryotic cell, we employed the well-established 7PA2 cell-based system that produces biologically active Aβ oligomers [38]. Using a sandwich-based approach, both mAB-O and mAB-M detected the oligomers derived from the 7PA2 cell system and this suggested that exposure of the N-terminal Aβ epitope is conserved between the *in vitro* and *in vivo*-derived oligomers (Fig. 8B). Similar results were also obtained using the recently described yeast-based system that expresses Aβ_1–42_ [39] (data not shown).

## Discussion

It has been more than a decade since the publication of the first report of cognitive improvement in a mouse model for AD as a result of passive immunization [40]. Due to the possible beneficial effect of adopting the same rationale on humans, these initial findings led to massive efforts from the pharmaceutical industry. Today more than ten clinical trials are in progress [41], [42]. Unfortunately, only modest cognitive improvement has so far been noted in humans, and individuals treated with the highest antibody titres have a significantly increased risk of developing vasogenic oedema and cerebral microhemorrhage [43]. There is, therefore, a need to better understand the process of passive immunization and acquire more effective antibodies having fewer side effects. Interestingly, passive vaccination is currently also emerging as an approach for other misfolding disorders including PD, amyotrophic lateral sclerosis, and pathologies associated with tau assemblies [44]–[51]. This further emphasizes the need for a general method to obtain antibodies with optimal properties.

Both cellular and animal models have shown that the fraction of small oligomeric assemblies – either preceding amyloid formation or representing a stand-alone entity formed in parallel with the fibrils – exert the most potent detrimental physiological effects [3], [4], [11] [2]. Consequently, these oligomeric assemblies represent a very interesting target for intervention. Intriguingly, antibodies have been developed that selectively identify conformational epitopes that are exclusively exposed in the oligomeric form [11], [13], [14], [18]–[27]. These antibodies provide an advantage compared to traditional antibodies because they do not react with the fibrillar or the monomeric forms, including putative precursor molecules, and, therefore, achieve a higher effective concentration toward the intended target. Unfortunately, the molecular properties associated with oligomer-specific antibodies are not well understood, and their development depends on stochastic events that hamper the targeted design and optimisation of these antibodies.

In the present work, we unveil features that render antibodies specific for oligomers and show how different properties affect their efficacy. The technique is easily adapted but, as we describe, several factors to be considered and extreme binding properties might be required to reach full capacity. Identifying these parameters has important therapeutic implications because highly efficient therapeutic antibodies can now be designed in detail.

The procedure is essentially divided into two steps in which a linear epitope, exposed within the monomeric and oligomeric forms but buried within the fibrillar state, can be combined with the effect of avidity to select for oligomer-specific binding properties. Identification of an epitope exposed within the oligomeric and monomeric state but buried within the fibrillar architecture is comparatively straightforward due to the significant structural differences that usually exist between oligomers and fibrils.

After selecting for impaired binding to the fibrils, the problem is reduced to discriminating between monomers (including precursor molecules) and oligomers. In this work, we show that even a simple divalent interaction can increase the affinity 1500 times compared to a monovalent interaction.

At first glance, this rationale implies that all antibodies having a valency higher than one would prefer binding to a multivalent target and, therefore, would be oligomer specific. This is, however, an oversimplification, and although a divalent interaction to a multivalent antigen causes a significant increase in binding strength this work describes how additional parameters in the binding need to be considered to achieve the desired oligomer-specific effect.

According to the fundamental properties of molecular binding, the level of saturation between a receptor and a ligand is always dependent on concentration. This implies that the concentration where oligomeric assemblies can be captured while the level of monovalent binding is maintained at insignificant levels is only valid within a certain range. This notion is of central importance because it implies that although the potentiating factor of avidity might be identical between high- and low-affinity antibodies their effective concentration range will differ. This range is dependent on the monovalent affinity as well as the potentiating factor due to the effect of avidity. Importantly, lowering the monovalent affinity will expand the effective concentration range. This is indicated in Figure 2, but to better illustrate the relative difference Figure 4B shows the concentration range for all of the antibodies, created in this work, in a non-logarithmic manner. Note in particular the large difference between mAB-O and mAB-M that both qualify as being oligomer specific according to the widely used dot blot technique shown in Figure 4A emphasizing why dot blot to discriminate between oligomers and monomers should be used with great care and always be accompanied with a technique where saturation levels can be monitored.

Because oligomer-specific antibodies have significant therapeutic potential, it is interesting to relate concentrations to therapeutic antibody levels. This has been the most well established with regard to AD, and commonly used serum concentrations of therapeutic antibodies targeting Aβ frequently range from 20 nM to 250 nM. Note that within this range most traditional antibodies display a very high level of monovalent saturation, and our results suggest that essentially only antibodies having very low monovalent affinities, such as mAB-O, fulfil the requirements of a selective binding within this concentration range. The monovalent K_D_ of mAB-O, corresponding to 36 µM is several thousand-fold higher than most traditional antibodies.

In this work we further expose how the efficacy by which antibodies find the thermodynamically most stable interaction (binding to the oligomer) is dependent on binding kinetics. We also illustrate how a slow rate of turnover, as clearly shown by ASyM, impairs the ability to bind the oligomeric target in the presence of the monomeric counterpart. Notably, the rate of turnover is also reflected within the dot blot analysis where only the high-affinity ASyM antibody remains attached to a monovalent target further emphasizing why dot blots should be used with care and should always accompanied by a complimentary technique.

Low-affinity binding might intuitively be associated with low specificity. This assumption is, however, not correct and can be explained using the thermodynamics of binding energies that have been described in detail by Kawasaki and co-workers [52]. In brief, the specificity of the binding is determined by the relative contribution of hydrophobic and polar interactions. A large contribution of hydrophobic interactions relative to polar interactions generally decreases the specificity. Because oligomers and protofibrils in general expose a large proportion of hydrophobic areas to the solvent, antibodies having a high proportion of the hydrophobic component might interact with these antigens in a rather nonspecific manner. A high proportion of hydrophobic interactions might, when combined with the concept of avidity, provide a possible molecular explanation for some of the previously reported generic oligomer-specific antibodies.

The antibodies presented within this work are all directed at comparatively hydrophilic areas, and their binding is, therefore, less likely to contain a high hydrophobic component. None of the antibodies presented within this work have been seen to cross react with other oligomers.

Because the binding specificity is determined by the choice of linear epitope, the importance of verifying a correct target should be stressed. We pinpoint putative structural discrepancies between *in vivo* and *in vitro*-derived assemblies of both Aβ and α-synuclein. Although the methods of sample preparation might affect epitope exposure, great care should be taken when choosing the epitope of binding and the initial screening should preferably be performed on *ex vivo*-isolated material under non-denaturing conditions.

Obtaining antibodies with high K_D_ values is a central issue, and in the random generation of hybridomas this is usually a stochastic event. Due to the generally lower level of affinity maturation within the murine immune system compared to, for example, that in rabbits [53], this appears to be fairly easy to accomplish in mice. We have not been able to successfully perform the corresponding approach in rabbit due to the significantly higher affinity of the generated antibodies.

Through the spectrum of different antibodies having vastly different binding properties, present work describes the proof of concept of a method to acquire oligomer-specific antibodies. The design of fully optimized antibodies e.g. for therapeutic purpose, is however a challenge for the future where importantly the monovalent affinities should be adjusted to the intended therapeutic concentration and where the cryptic epitope exclusively buried within the fibrils should be chosen with great care. We explicitly expose the possibility of applying our technique to the IgG isotype which is amenable to recombinant techniques and presents an essentially unlimited potential for variability that facilitates production of antibodies having the desired oligomer-binding properties. This possibility essentially also eliminates the dependence on developing antibodies within an animal, which is an often unpredictable and stochastic process, because optimization of affinity can now instead be directly performed on pre-existing cell lines and sequences from known antibodies using the versatile techniques available for genetic modification and recombinant expression of antibodies.

The approach may also be combined with other features e.g. specific reactivity toward modifications such as the clinically important pyroglutamate modification of the Aβ peptide [54] or the choice of an IgG subtype that can minimise unwanted Fcγ receptor-mediated activation of microglia cells [55]. The technique also provides opportunities to optimize properties of already existing monoclonal antibodies, and the same approach could also be used for the design of antibodies that exclusively target the fibrillar form if a corresponding epitope only exposed on fibrils but not oligomers, could be detected.

In conclusion, we show how the effect of avidity from a divalent antibody in combination with the choice of a cryptic epitope, exclusively buried within the fibrillar form, can be used to acquire highly oligomer-specific antibodies. We demonstrate how the monovalent affinity can be used as a parameter to modulate the oligomer-specific properties and that a decrease in monovalent affinity dramatically increases the concentration range over which oligomers and monomers can be efficiently discriminated. We also show that high affinity antibodies, having a low turnover rate, becomes kinetically blocked and impaired to identify the oligomeric fraction in a stoichiometric excess of the monomeric counterpart. The presented approach has been demonstrated by the production of antibodies that target oligomeric assemblies of Aβ and α-synuclein. However, the method is universal and has translational implications for diagnostic and therapeutic efforts regarding all protein misfolding disorders associated with oligomeric and fibrillar assemblies.

## Methods

### Immunisation and generation of monoclonal hybridomas

Mice (BALB/c, female, 8–10 weeks old) were immunised with either Aβ_1–42_ (Alexotech AB, Umeå, Sweden) or the full-length sequence of human α-synuclein (Alexotech AB). All immunisations were performed by Agrisera AB (Vännäs, Sweden). Generation of hybridomas was performed by Swedclone AB (Umeå, Sweden). Initial screening of clones and determination of the binding site was accomplished using standard ELISA techniques where all peptides were directly immobilised on the surface. Peptide fragments Aβ_1–10_, Aβ_1–16_, Aβ_1–20_, Aβ_3–10_, and Aβ_3–40_ were purchased from BioNordika (Stockholm, Sweden). A library consisting of 15mer peptides synthesised with a five-residue overlap covering the human α-synuclein protein sequence was obtained from Innovagen (Lund, Sweden).

### Preparation of Aβ oligomers

Recombinant Aβ_1–42_ (Alexotech AB) was dissolved in 20 mM NaOH followed by addition of a 10× stock solution of PBS and adjustment of the pH to 7.4 using HCl. The final Aβ concentration was 100 µM and the sample was incubated for 1 hour at room temperature (RT) with slow agitation. Isolation of oligomers having an apparent molecular weight between 25 kDa and 150 kDa, as determined by a molecular weight standard (Bio-Rad, 151-1901), was performed by size-exclusion chromatography (SEC) (GE Superdex-G200 10/300) at 4°C. The concentration of all samples was determined by absorbance measurements at 280 nm. Yeast-derived oligomers were prepared according to [39].

### Preparation of α-synuclein oligomers and fibrils

α-synuclein oligomers were made as described previously but with minor modifications [56]. Briefly, lyophilised α-synuclein (Alexotech AB) was dissolved in 10 mM sodium phosphate buffer (pH 7.4). Dopamine was added to generate a final concentration of 73 µM α-synuclein and 1 mM dopamine. The sample was incubated for 24 hours at 37°C with agitation and separated by SEC (GE Superdex-G200 10/30, Uppsala, Sweden) in PBS. This approach results in a stable oligomeric conformation having an apparent molecular weight around 600 kDa, shown in supporting information Figure S3. Preparations were aliquoted and kept frozen until use. Fibrillar α-synuclein was induced through prolonged incubation at 10 mg/mL in PBS at 37°C, collected by centrifugation, and washed once in PBS.

### Dot blot analysis of Aβ fibrils, oligomers, and monomers

To compare the ability of the different antibodies to discriminate between oligomers and fibrils, a specific oligomer fraction from the sample described above was split into two parts where one part was subjected to incubation at 37°C for 3 days to induce fibrils and the remaining sample was frozen at −20°C. Uniform presence of fibrils was verified using native PAGE and AFM. Equal amounts of Aβ fibrils and oligomers were applied to a nitrocellulose membrane and air dried. The membrane was blocked using 5% non-fat milk and incubated with the corresponding antibodies (25 nM) for 1 hour. All washes of the membrane were done using PBS containing 0.25% Tween-20 (PBST). Bound antibodies were detected using an anti-mouse HRP-conjugated secondary antibody at a 1∶1500 dilution (GE Healthcare, Uppsala, Sweden). Detection of the polyclonal OC antibody was performed with an anti-rabbit HRP-conjugated secondary antibody (GE Healthcare). Detection used the ECL prime western blotting detection reagent (GE Healthcare).

Dot blot analysis of α-synuclein was performed according to the same procedure where equal amounts of α-synuclein fibrils, oligomers, and monomers were applied to a nitrocellulose membrane and air dried. The membrane was blocked using 5% non-fat milk and incubated with the corresponding antibodies (2 µg/mL) for 1 hour followed by washing in PBST. Bound antibodies were probed using an anti-mouse HRP-conjugated secondary antibody at 1∶1500 dilution (GE Healthcare).

### Affinity determination in solution using competitive ELISA

To illustrate the potentiation in binding strength of a bivalent interaction occurring in solution, we have used a simple approach where oligomers and antibodies are probed in solution before analysis. The setup is based on mixing a constant concentration of antibody with various concentrations of oligomers ranging from a substoichiometric ratio to a molar excess of oligomers. The levels of unbound antibodies can be measured, and the concentration at which 50% of the antibodies are bound reflects the binding affinity.

In brief, a fixed concentration of antibody was dissolved in PBS with 0.005% Tween and BSA (1 mg/mL) at RT. Different concentrations of oligomers were added to the antibody solution in separate tubes and incubated until equilibrium was reached. These samples were subsequently transferred to an ELISA plate onto which the corresponding oligomers had been immobilized. Antibodies not bound to oligomers in solution could now instead bind to oligomers on the plate and thus be quantified according to standard ELISA techniques using a secondary anti-murine antibody (GE Healthcare). Thus only free antibodies – those not bound to the oligomers in solution in the initial step – are detected. The antibody concentrations were quantified by preparing a standard curve of known concentrations of antibody alone that binds to the ELISA plate. From the standard curve, it is possible to determine the amount free antibody in the oligomer samples and thus determine the amount of antibodies bound to the oligomers.

A binding curve was generated by plotting the concentration of bound antibody to the oligomer against the concentration of free oligomers as described in Equation 1 where B_max_ is the maximum binding plateau, X is the free concentration of Aβ oligomers, and K_D_ represents the concentration at which 50% of the divalent antibodies are bound to the oligomers.
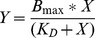
(1)


We show that the potentiating effect as a result of divalent binding is between 600 and 1500 fold. This strong potentiation creates a technical limitation, and affinity determination can only be performed if the divalent K_D_ is within the range of detection. This prevents the determination of binding affinity of high-affinity antibodies and, therefore, the divalent K_D_ of ASyM could not be determined.

### Binding theory describing affinity and avidity

The affinity (K_D_), which represents the strength of a single receptor-ligand interaction, is determined by the association rate of receptor and ligand (k_on_) and the dissociation rate of the complex (k_off_) according to Equation 2.
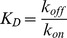
(2)K_D_ can be converted to free energy (ΔG) according to Equation 3 where R is the general gas constant of 1.985 cal/K, T is the temperature in Kelvin, and K_A_ is the inverse of K_D_.
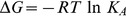
(3)


Avidity is the combined synergistic strength of binding affinities when several interactions occur simultaneously. This means that the total binding strength is increased by the sum of the interactions but is reduced by the loss of entropy as a result of restriction in free motion. Regarding IgG binding, the maximum valence of simultaneously occupied sites is two. The difference between affinity (one interaction) and avidity (in the case of IgG – a maximum two interactions) can be described theoretically. The free energy can be separated into two opposing energies, one favouring association and one opposing it. This is illustrated in Equation 4 where ΔG_bond_ is the free energy of all chemical forces contributing to the association and ΔG_s_ is the free energy required to immobilise one subunit in the binding [57].
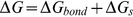
(4)


Free energy is required in a protein-protein interaction due to the entropy loss caused by immobilisation of one of the proteins. Before binding, each subunit has three degrees of rotational freedom and three degrees of translational freedom. In a complex, one subunit still maintains its three degrees of freedom whereas the other protein loses its ability to move.

When determining the free energy for a multivalent antibody∶oligomer interaction, ΔG_bond_ is counted once for every binding interaction. For an IgG antibody with two binding sites, ΔG_bond_ will thus be counted twice. ΔG_s_, on the other hand, will only be counted once because the oligomer is already immobilised after the first interaction and the second interaction takes place without loss of entropy. In reality, there is also a small loss of entropy in the second interaction when the second fragment antigen binding (Fab) region loses its degrees of rotational freedom. The second interaction can also be inhibited by structural constraints depending on immobilisation of the first interaction.

### Competition ELISA to probe the efficacy of oligomer binding in the presence of monomers

Purified oligomers were coated onto Nunc-Immuno MaxiSorp plates (Nunc, Roskilde, Denmark) at a concentration of 5 µg/mL in PBS followed by blocking of unbound sites using 5% non-fat milk dissolved in PBS. Bound murine antibodies were mixed with a titration of their corresponding epitope in a stoichiometric ratio as indicated in Figure 3 and were evaluated for their ability to bind the immobilised oligomers in the presence of the monomeric peptides corresponding to their epitope. Binding of mAB-O and mAB-M was competed using different ratios of Aβ_1–10_. ASyO2 binding was competed using the α-synuclein Glu131–Ala140 peptide; ASyO2 binding was competed using the α-synuclein Gly111–Tyr125 peptide; and ASyM was competed using the α-synuclein Met1–Val15 peptide. The complexes were incubated for 1 hour, and unbound antibodies were removed through repeated washing with PBS containing 0.25% Tween-20. Bound murine antibodies were probed using HRP-conjugated anti-mouse antibodies diluted 1∶1500 in blocking buffer (GE Healthcare) and quantified with EC-blue (Medicago, Uppsala, Sweden).

### Immunohistochemistry

Sectioned paraffin-embedded tissue samples from human AD hippocampus or PD midbrain were de-waxed and rehydrated in an ethanol gradient. Antigens were retrieved in sodium citrate buffer (pH 6) at 95°C for 1 h. The tissue sections were separately incubated for 1 h at RT with rabbit polyclonal anti-Aβ (AS328, Agrisera), mAB-O, or mAB-M antibodies for AD hippocampus sections or with rabbit polyclonal anti-α-synuclein (AS08-358, Agrisera), ASy-O2, or ASyO5 antibodies for the PD tissue samples. The immunoreactivity was detected with the anti-mouse or anti-rabbit IgG Peroxidase Reagent Kit (ImmPRESS, Vector Laboratories, Inc.) followed by developing with the ImmPACT AEC Peroxidase Substrate kit (Vector Laboratories, Inc.).

### Cytotoxicity assay

SH-SY5Y neuroblastoma cells were obtained from the European Collection of Cell Cultures (Centre for Applied Microbiology and Research, Wiltshire, UK). Cells were cultured as described in [58].

SEC, performed directly in MEM medium without supplementary factors, was used for specific isolation of the oligomeric fraction and removal of any remaining monomeric material. Oligomers were mixed with the antibody at an Aβ∶antibody ratio of 5∶1 and different amounts of Aβ_1–10_ (Anaspec, Freemont, CA, USA). Cell viability was measured after 48 hours using a resazurin reduction test [59]. Cytotoxicity is presented as a percentage of the fluorescence of control cells incubated with MEM only. Statistical analysis was performed using GraphPad Prism version 5.01 for Windows (GraphPad Software, San Diego, CA, USA). Viability of cells incubated with Aβ oligomers in the presence or absence of mAB-O and Aβ_1–10_ was analysed using one-way ANOVA with Dunnett's post-test. Statistical significance was set to a p-value≤0.001.

### Detection of α-synuclein oligomers in CSF using sandwich ELISA

The ASyO2 antibody was coated onto black ELISA plates (Nunc-Immuno MaxiSorp plates, Nunc). Non-specific sites were blocked with 2% non-fat milk dissolved in PBST. CSF samples from 10 different individuals having a clinically verified PD diagnosis as well as 10 age-matched controls were applied in triplicate. The CSF samples were incubated for 2 h at RT with slow agitation. Unbound material was removed through extensive washing with PBST. Bound oligomers were identified using biotin-labelled ASyO2 antibody. After subsequent washing with PBST, bound antibodies were detected using Ultra Streptavidin-HRP (Nordic Biolabs, Stockholm Sweden) and visualized using chemoluminescence (Supersignal Pico, Nordic Biolabs).

### Detection of Aβ oligomers derived from the 7PA2 cell line using sandwich ELISA

mAB-O and mAB-M were coated onto an ELISA plate in PBS. Nonspecific sites were blocked with 2% non-fat milk dissolved in PBST. Conditioned medium from the well-established 7PA2 cell line was prepared as described in [38] and applied to the immobilised antibodies for 1 hour at 4°C. Bound Aβ was detected using a biotin-labelled form of the previously described OMAB antibody [29] (Agrisera) that binds to the same epitope (Ala3–Ser8) as both mAB-O and mAB-M. After subsequent washing with PBST, bound antibodies were detected using Ultra Streptavidin-HRP (Nordic Biolabs) and visualized using chemluminescence (Supersignal Pico, Nordic Biolabs).

### Ethics statement

All animal experiments were approved by the Umeå Ethical Board of Animal Research and were performed according to the Declaration of Helsinki (permit number A47-07). Collection and analysis of human samples was approved by the Ethical Committee of Umeå University and the Regional Ethical Review Board in Umeå, section for Medical Research (approval number 94-135, 19941014), and adhered to the principles of the Declaration of Helsinki. The information about the project was given both orally and in written form, and a signed copy was left in the hospital files. After the death of a patient, the next of kin must give his or her consent for brain tissue to be used (this cannot be given prior to the demise of the patients according to our ethical permit and the standard operating procedures at our hospital). Given the large distances in our hospital's area of responsibility (600 square km), it is not possible for the physician to meet the next of kin in person to obtain the written consent. However, the information to make the consent informed was duly noted in the hospital files with a signed copy, i.e. the next of kin was informed and gave his or her acknowledgement of this in writing. This information as well as the date and time of the phone call of the consent for the autopsy were noted in the hospital files according to a procedure approved by the Ethical Committee.

## Supporting Information

Figure S1 **Atomic Force Analysis of Aβ and α-synuclein.** An aliquot of each sample was diluted in water to approximately 500 nM and applied to freshly cleaved ruby red mica (Goodfellow, Cambridge, UK). All samples were allowed to adsorb for 30 s. The mica was then washed with distilled water three times and air-dried. Analysis was performed using a Nanoscope IIIa multimode AFM™ (Digital Instruments Santa Barbara, USA) in tapping mode in air. A silicon probe was oscillated at approximately 300 kHz and images were collected at an optimized scan rate corresponding to 1–4 Hz. Scale bar = 500 nm (A) Aβ1–42 monomer. (B) Aβ1–42, oligomers. (C) Aβ1–42, fibrils. (D) α-synuclein, monomer. (E) α-synuclein, oligomers. (F) α-synuclein, fibrils.
(TIF)

Figure S2 **SPR analysis of the monovalent interaction.** Antibodies were immobilised at a density of 10 000–15 000 RU on a CM5 chip (GE Healthcare) using standard amine-coupling chemistry at pH 5. Determination of monomeric affinity constants for the anti-Aβ antibodies was performed using either Aβ(1–40) or Aβ(1–16), in PBS buffer, at a flow rate of 50 µl/min in at 25°C. SPR sensograms were corrected for non-specific interactions to a reference surface, and by double referencing. The affinity constants for ASyM, and ASyO2 were performed in a similar manner using either full-length α-synuclein or the monomeric peptide fragment covering the epitope of the specific antibody. The dissociation constant was determined by fitting the response at the end of each of the association phases to a single-site binding isotherm. SPR sensograms acquired through probing immobilised antibodies towards their corresponding monovalent antigens as described within material and methods. (A) mAB-M. (B) mAB-O. (C) ASyM. (D) ASyO2. (E) ASyO5.
(TIF)

Figure S3 **Size exclusion chromatography for isolation of α-synuclein oligomers.** Lyophilised α-synuclein was dissolved at 10 mg/ml in 10 mM sodium phosphate buffer (pH 7.4). Dopamine was added to generate a final concentration of 73 µM α-synuclein and 1 mM dopamine. The sample was incubated for 24 hours at 37°C with agitation and separated through size exclusion chromatography (GE Superdex-G200 10/30, Uppsala, Sweden) in PBS. The fractions within the borders separated by the striped lines where used.
(TIF)
