# Supplementary figures and images for: A Generic Method for Design of Oligomer-Specific Antibodies
Source: PLoS One. 2014 Mar 11;9(3):e90857. doi: 10.1371/journal.pone.0090857 (PMC3949727; doi:10.1371/journal.pone.0090857)

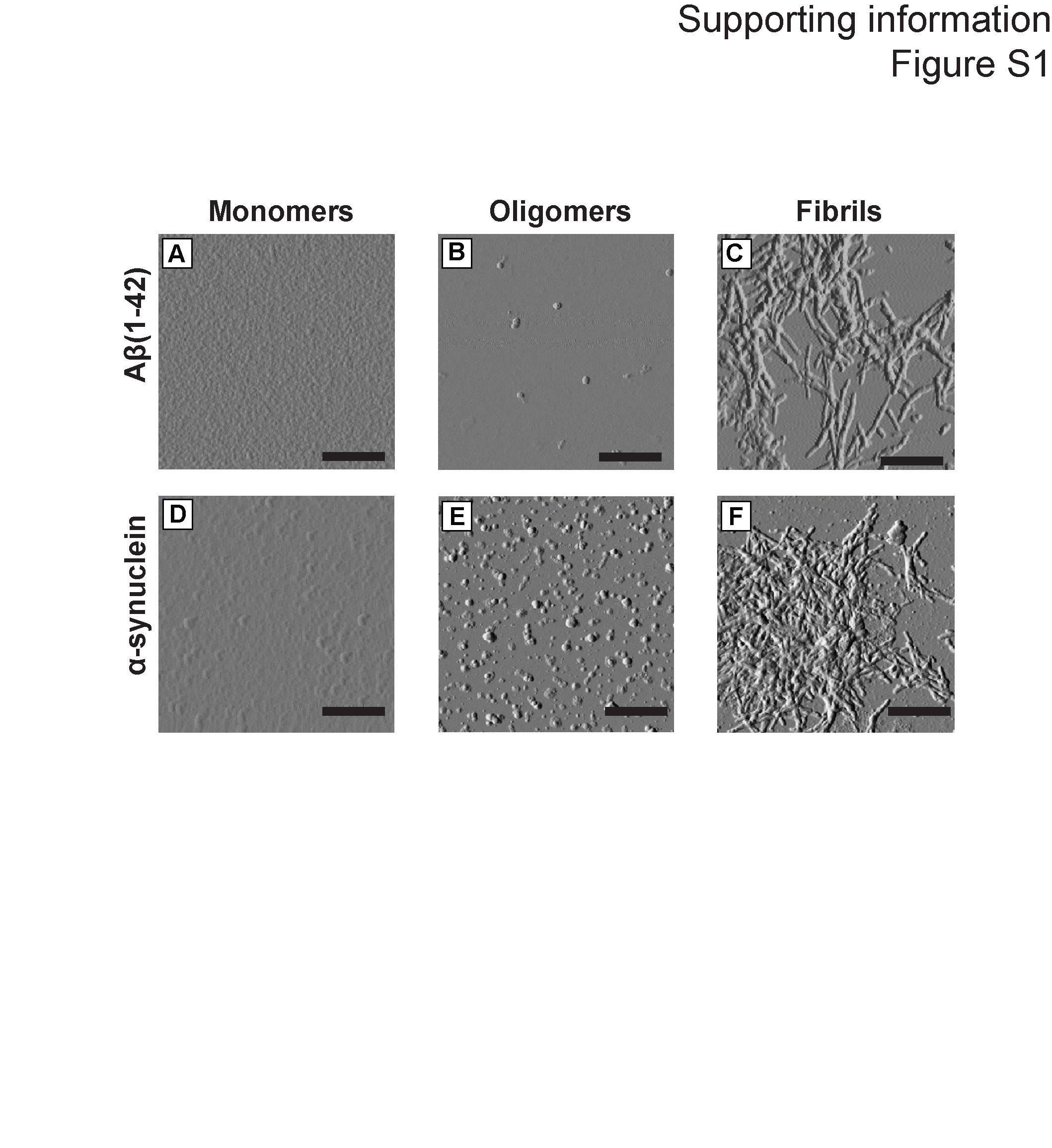

Supplement: Figure S1 — Atomic Force Analysis of Aβ and α-synuclein. An aliquot of each sample was diluted in water to approximately 500 nM and applied to freshly cleaved ruby red mica (Goodfellow, Cambridge, UK). All samples were allowed to adsorb for 30 s. The mica was then washed with distilled water three times and air-dried. Analysis was performed using a Nanoscope IIIa multimode AFM™ (Digital Instruments Santa Barbara, USA) in tapping mode in air. A silicon probe was oscillated at approximately 300 kHz and images were collected at an optimized scan rate corresponding to 1–4 Hz. Scale bar = 500 nm (A) Aβ1–42 monomer. (B) Aβ1–42, oligomers. (C) Aβ1–42, fibrils. (D) α-synuclein, monomer. (E) α-synuclein, oligomers. (F) α-synuclein, fibrils. (TIF) [file pone.0090857.s001.tif]

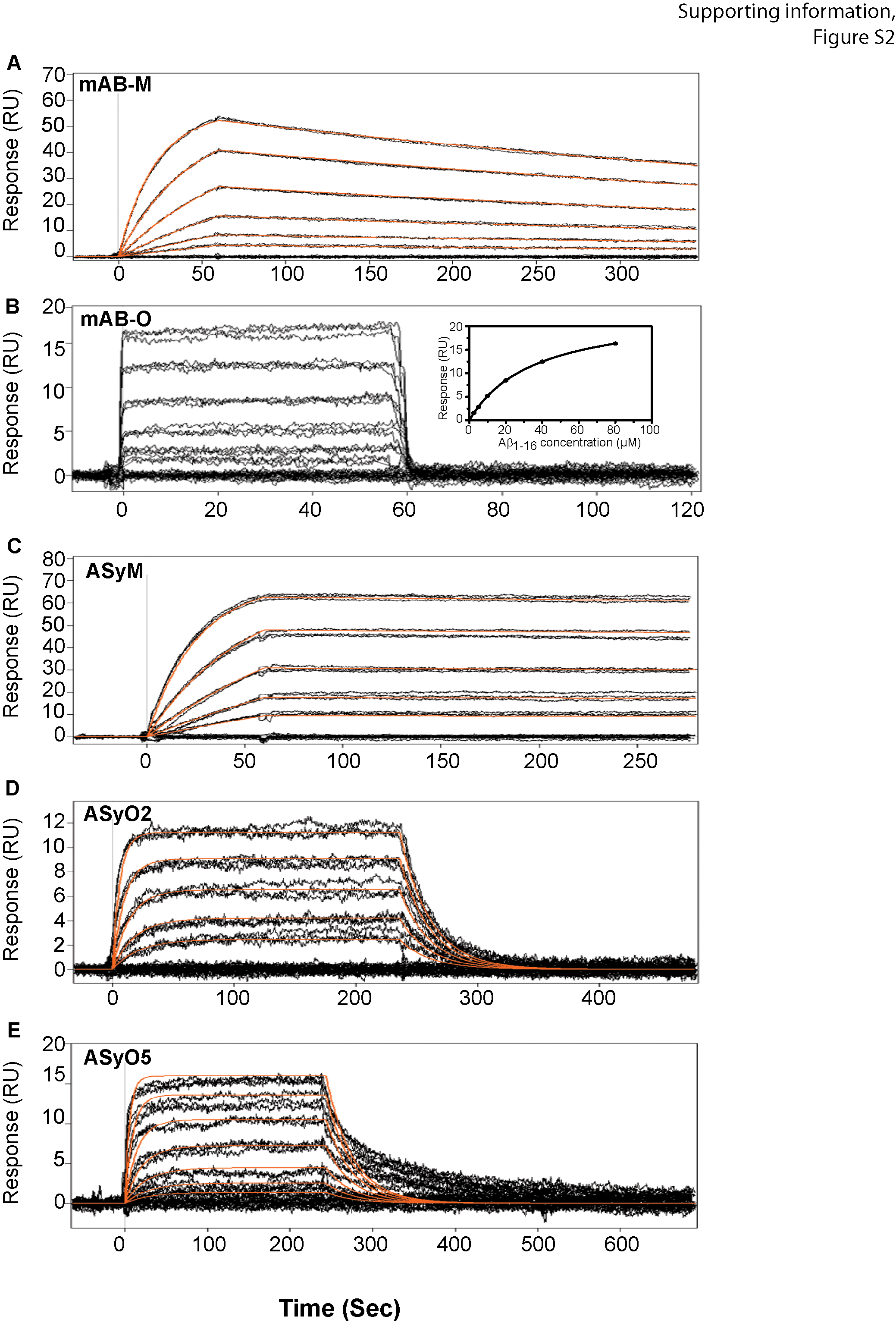

Supplement: Figure S2 — SPR analysis of the monovalent interaction. Antibodies were immobilised at a density of 10 000–15 000 RU on a CM5 chip (GE Healthcare) using standard amine-coupling chemistry at pH 5. Determination of monomeric affinity constants for the anti-Aβ antibodies was performed using either Aβ(1–40) or Aβ(1–16), in PBS buffer, at a flow rate of 50 µl/min in at 25°C. SPR sensograms were corrected for non-specific interactions to a reference surface, and by double referencing. The affinity constants for ASyM, and ASyO2 were performed in a similar manner using either full-length α-synuclein or the monomeric peptide fragment covering the epitope of the specific antibody. The dissociation constant was determined by fitting the response at the end of each of the association phases to a single-site binding isotherm. SPR sensograms acquired through probing immobilised antibodies towards their corresponding monovalent antigens as described within material and methods. (A) mAB-M. (B) mAB-O. (C) ASyM. (D) ASyO2. (E) ASyO5. (TIF) [file pone.0090857.s002.tif]

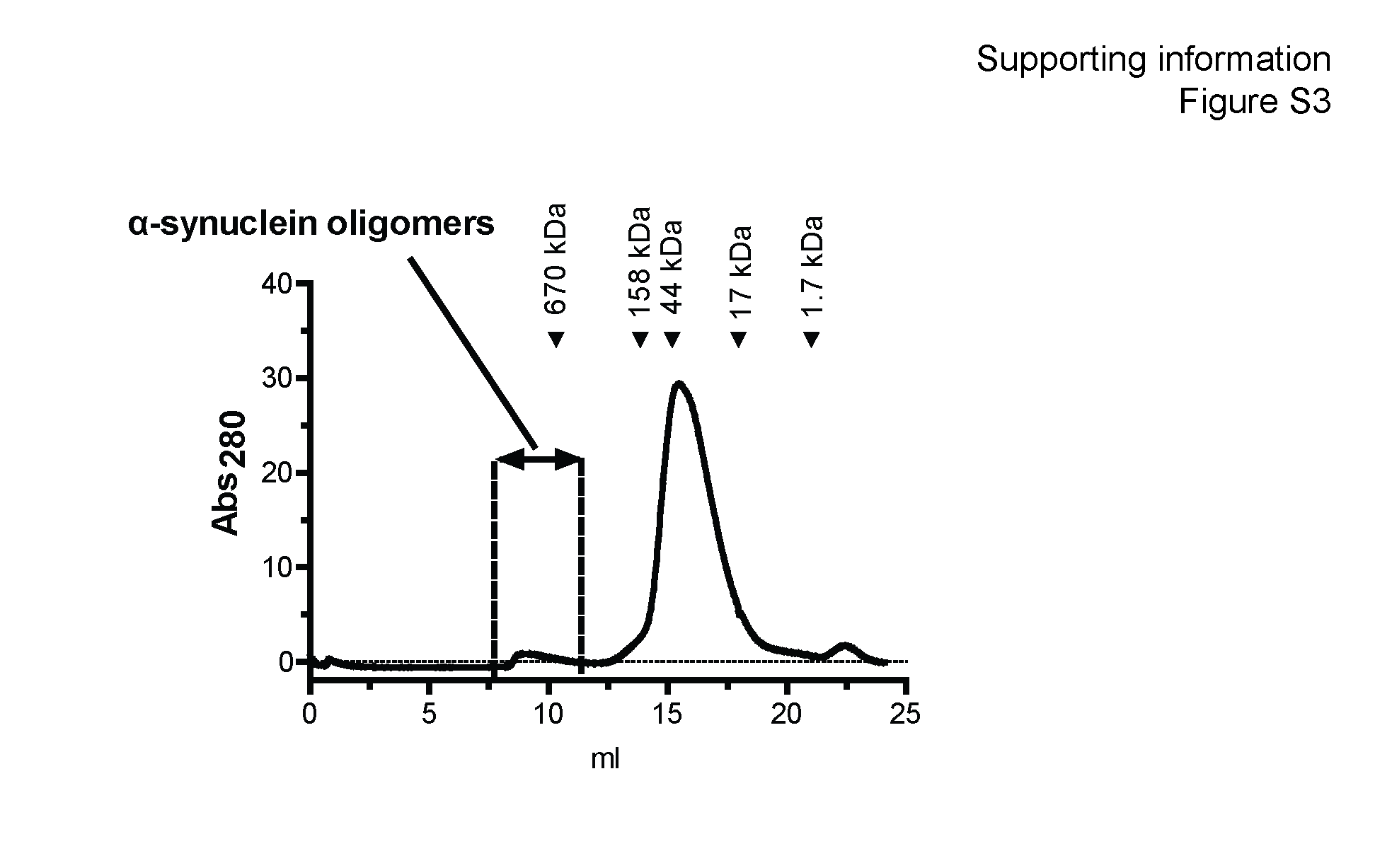

Supplement: Figure S3 — Size exclusion chromatography for isolation of α-synuclein oligomers. Lyophilised α-synuclein was dissolved at 10 mg/ml in 10 mM sodium phosphate buffer (pH 7.4). Dopamine was added to generate a final concentration of 73 µM α-synuclein and 1 mM dopamine. The sample was incubated for 24 hours at 37°C with agitation and separated through size exclusion chromatography (GE Superdex-G200 10/30, Uppsala, Sweden) in PBS. The fractions within the borders separated by the striped lines where used. (TIF) [file pone.0090857.s003.tif]
